# Can discrete event simulation be of use in modelling major depression?

**DOI:** 10.1186/1478-7547-4-19

**Published:** 2006-12-05

**Authors:** Agathe Le Lay, Nicolas Despiegel, Clément François, Gérard Duru

**Affiliations:** 1 Laboratoire d'Analyse des Systèmes de Santé, Université Claude Bernard, Lyon 1, France; 2 International Health Economics and Epidemiology Department, H. Lundbeck A/S, Paris, France

## Abstract

**Background:**

Depression is among the major contributors to worldwide disease burden and adequate modelling requires a framework designed to depict real world disease progression as well as its economic implications as closely as possible.

**Objectives:**

In light of the specific characteristics associated with depression (multiple episodes at varying intervals, impact of disease history on course of illness, sociodemographic factors), our aim was to clarify to what extent "Discrete Event Simulation" (DES) models provide methodological benefits in depicting disease evolution.

**Methods:**

We conducted a comprehensive review of published Markov models in depression and identified potential limits to their methodology. A model based on DES principles was developed to investigate the benefits and drawbacks of this simulation method compared with Markov modelling techniques.

**Results:**

The major drawback to Markov models is that they may not be suitable to tracking patients' disease history properly, unless the analyst defines multiple health states, which may lead to intractable situations. They are also too rigid to take into consideration multiple patient-specific sociodemographic characteristics in a single model. To do so would also require defining multiple health states which would render the analysis entirely too complex. We show that DES resolve these weaknesses and that its flexibility allow patients with differing attributes to move from one event to another in sequential order while simultaneously taking into account important risk factors such as age, gender, disease history and patients attitude towards treatment, together with any disease-related events (adverse events, suicide attempt etc.).

**Conclusion:**

DES modelling appears to be an accurate, flexible and comprehensive means of depicting disease progression compared with conventional simulation methodologies. Its use in analysing recurrent and chronic diseases appears particularly useful compared with Markov processes.

## I. Background

Depression is a widespread condition associated with significant functional and social deterioration as well as extensive direct and indirect health care costs. A recent review of epidemiological studies estimates the annual prevalence rate of major depression at approximately 5% in Europe [1]. Within the next 20 years, depression is predicted to become one of the leading causes of disability worldwide [2].

In 2001, the National Institute of Mental Health authorized additional research on preventing relapse in major depression as a part of a larger effort to find effective treatments capable of producing long-term durable recovery [3]. Depression is a recurrent, potentially chronic and disabling condition. Acute treatments for depression, although effective, are often not sufficient enough for a large percentage of patients in preventing either subsequent functional impairment due to residual symptoms, or recurrent episodes. The primary objective of an intervention to prevent relapse is sustained remission of depressive symptoms. However, it is increasingly accepted that economic considerations need to be taken into account. Rising costs of interventions along with newer and more expensive antidepressant treatments bring up to questions about the cost-effectiveness of therapeutic interventions. Economic evaluation can assist decision-makers by providing additional support in making informed judgments concerning the allocation of increasingly scarce healthcare resources [4].

Quantifying the economic implications of a healthcare intervention requires precisely defining the target population, the characteristics of the disease and the therapeutic intervention. It also requires structuring the possible trajectory of patients in a logical, realistic order over time by considering the events that may occur, together with their health and economic implications. Providing a computational framework to illustrate disease progression over time as accurately as possible is necessary. Decision trees have been used successfully despite general recognition that they have severe limitations when applied to medical conditions [5]. Markov models provided an alternative that allowed analysts to picture the course of a disease in terms of mutually exclusive health states and the transitions among them. While this technique considers time more explicitly and can be analyzed very efficiently, Markov models are considered highly rigid, mainly because of the lack of 'memory' imposed by the stochastic process.

Discrete Event Simulation (DES) models might offer a natural way of adequately depicting patient disease course throughout the health system [6] by making it possible to take into account important (baseline) prognostic factors together with life events interactions.

In this case study, our intention was to identify and compare the strengths and limits of discrete event simulation models with those of Markov models in portraying depression dynamics. To achieve this goal we employed a three-step process:

1. We described the clinical features specific to unipolar major depression.

2. We conducted a conceptual implementation of a Markov model and a DES model to detect possible abilities to address disease-specific issues relevant to major depression.

3. We discussed and compared the ability of each type of model to adequately reflect disease progression over time.

## II. Key clinical features of unipolar depression

MB. Keller and colleagues reviewed important factors predisposing patients to recurrence of depressive symptoms and highlighted several risk factors that should be considered when modelling disease evolution [7].

### II.1 Definitions

Concentrating on prophylactic strategies requires consensus of definitions for specific concepts such as *relapse *and *recurrences *of depressive symptoms. In 1988, the MacArthur Foundation Research Network on the Psychobiology of Depression consensus group agreed on the definition of terms required to designate the relevant change points over the course of illness. These definitions have provided a framework for deciding what constitutes "an episode" and have further clarified the concepts of severity and duration.

• Remission is defined as "a relatively brief period during which an improvement of sufficient magnitude is observed so that the individual is asymptomatic, i.e. the patient no longer meets syndromal criteria for the disorder and has no more than minimal symptoms".

• Recovery is defined as "an asymptomatic period that lasts longer than the remission period". This definition is used to designate recovery from the episode, not from the depressive symptoms *per se*, and implies a sustained remission of symptoms.

• Relapse is defined as "the early return of depressive symptoms following an apparent remission".

• Recurrence is defined as "the appearance of a *new *episode of major depressive disorder and thus can only occur during a period of recovery".

**Figure 1:**
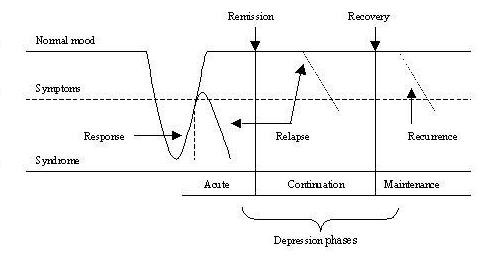
Diagram of the five possible outcomes across the three phases of treatment of depression (Source: Thase M.E., 2000). Acute phase: usually 2 to 3 months; Continuation phase: usually 4 to 6 months; Maintenance phase: can last up to 5 years (Definitions from Kupfer et al. study, 1992)

Figure 1 provides a visual understanding of how distinct phases of depression differentiate relapses from recurrences, and remission from full recovery.

In this paper, a "depressive event" is defined as the occurrence of depressive symptoms. A depressive episode may include several depressive events.

### II.2 Important risk factors

The following section highlights key features in terms of risk factors for unipolar major depression. The illustrative data presented hereafter were extracted from published literature [7-10]. Long-term prospective studies of patients with depression are somewhat scarce, therefore this work was mainly based on the National Institute of Mental Health (NIMH) Collaborative Program on the Psychobiology of Depression study [7,9,10]. This study was a prospective, naturalistic long-term follow-up that aimed to describe the episodic course of illness in major depressive disorder. Recruited individuals received either outpatient or inpatient care (outpatients represented 25% of the total sample).

**Figure 3:**
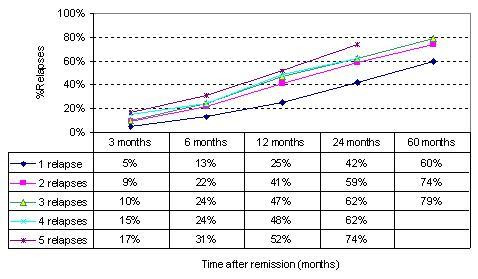
Cumulative probability of relapse after remission from depressive symptoms given the number of prior depression events (Source: Solomon et al., 2000).

One of the major findings from this long-term follow-up study suggested that the number of previous depressive events a patient experienced significantly influenced their probability of relapse (Figure 3).

**Figure 4:**
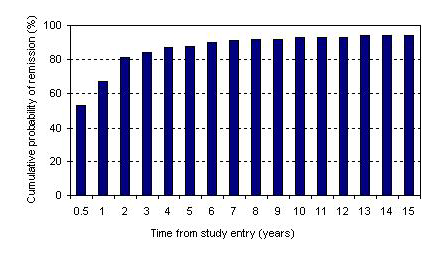
Cumulative probability of remission from index episode of depression (Source: Keller et al., 1998).

In approximately 20% of cases, as duration of depressive symptoms increased, the chances of remission decreased (Figure 4). These findings reinforce the chronic nature of the illness for a substantial number of patients [11].

The presence of residual depressive symptoms has also been proven to be associated with an increased risk of short-term relapse as well as with a long-term chronic course. Patients' attitude towards treatment has also been widely discussed as a key predictive factor of the long-term course of the disease. Olfson and colleagues [12] recently showed that approximately 4 out of 10 patients (42.4%) who initiated antidepressant treatment for depression discontinued the antidepressant medication during the first 30 days of treatment, and among those who continued antidepressant therapy for more than 30 days, one-half (52.1%) discontinued the medication during the subsequent 60-day period. Moreover, a 2-year naturalistic study showed superior long-term recovery in patients who were adherent to antidepressant medication compared with non-adherent patients [8,13].

Lastly, sociodemographic characteristics such as age and gender have also been proven to be significant factors to be taken into consideration [14].

**Figure 2:**
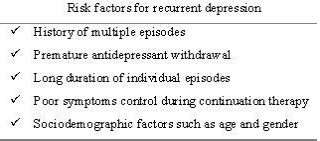
Key risk factors for recurrent depression

Figure 2 summarizes the key factors for recurrent depression.

Management of patients therefore requires differentiating between their sociodemographic characteristics, disease history, number of prior episodes and compliance to treatment over time.

## III. Application

Acceptance of the definitions proposed brings us to the comparison of two computational frameworks for modelling real-life disease evolution for in- and outpatients with major unipolar depression over a period of 10 years.

The final health outcomes of interest were time spent without depressive symptoms (i.e. time in remission and full recovery) and the number of relapses and recurrences occurring over the study period. The simulation models described hereafter illustrate disease progression over time regardless of therapeutic strategy and take into consideration realistic patient behaviour patterns as well as important prognostic factors. We provide additional technical details on both Markov and Discrete Event Simulation models, together with a practical example of both modelling methods. The software used to implement the simulation models was TreeAge Pro 2006 Healthcare Software, release 0.1 by TreeAge Software Inc., Williamstown, MA 01267 USA.

### III.1 Markov models

Markov modelling is a decision-analytic technique that characterizes the prognosis of a cohort of patients by assigning them to a fixed number of health states and then models transitions among health states [15]. Markov models (typically Markov chains) assume transition probabilities to be constant over time. However, it's possible to bypass this strict assumption by modelling non-homogeneous (i.e. time-dependent) Markovian stochastic processes. Markov models are particularly suited to modelling events of interest that occur repeatedly over a long period of time [5,15]. However, an important limitation of Markov models is that they lack "memory". This means that the probability of moving from one state to another does not take into account the history of the patient before he or she arrived in that state. This is also referred to as the Markovian assumption.

**Figure 5:**
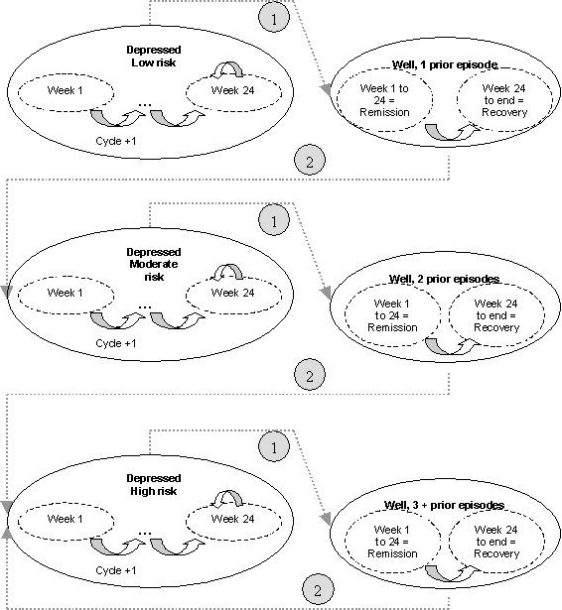
Markov model synthetic representation. Cycle length: 1 week. Circles represent health states. Dotted circles represent temporary health states. Arrows represent transition probabilities: 1: Weekly probabilities of remission, according to time spent with the illness. 2: Weekly probabilities of relapse according to patient's risk profile.

In our illustrative case (picture in Figure 5), health states were divided into three levels of risk -low, moderate, high (reflecting patient's number of previous episodes), each being divided into multiple temporary states associated with varying probabilities of remission according to the time elapsed in the disease state (in order to handle illness persistence issues). Therefore, for each level of risk, on the basis of a 1-week cycle (the accepted time span in MDD before observing any potential health transitions), we defined 24 temporary depressed states, i.e. 24 weekly remission probabilities adjusted for the duration of the disease. If the patient was still depressed at week 24, a constant probability of remission was applied. The number of temporary states was chosen according to the accepted management of an episode (i.e. a continuation period of 6 months) [16,17].

This resulted in 24*3 = 72 (temporary) health states accounting for risk levels and duration of the illness.

To differentiate remission periods from recovery periods (for increased precision when assessing the ability of a given strategy to further delay development of depressive symptoms), it was necessary to divide the "well" state into two separate temporary states. The first state was remission, i.e. the first 24 weeks following the disappearance of symptoms (in accordance with clinical guidelines which define a minimum of 24 symptom-free weeks before concluding that the patient has achieved full recovery). The second state was the full recovery period (i.e. a period of remission longer than 24 weeks).

The data required to specify this simulation model (in terms of clinical data exclusively) are survival distributions of remission and relapse at each cycle (i.e. one-week transition probabilities), conditional on the number of previous depression events. The time spent in the any health state may be summed over the period (i.e. 10 years = 520 cycles) and eventually discounted according to applicable rates.

This somewhat "simple" model (i.e. in terms of the number of risk factors taken into account) demonstrates the suitability of Markov models in addressing the key features of importance when modelling depression evolution over time. First, they handle the problem of patients' history of the disease by splitting health states according to different risk levels (low, moderate, high). This is computationally acceptable. Second, the chronic nature of the disease (for approximately 20% of patients, as mentioned previously) was managed at the expense of defining multiple health states (i.e. 72 states encoded as "tunnel" variables), making it possible to assign varying transition probabilities according to the time spent in the "depressed" state. Lastly, Markov models can distinguish between remission and recovery periods by using temporary states (i.e. 6 more health states). Therefore, a Markov representation of the problem requires defining at least 72+6 = 78 health states to properly take into consideration primary relevant risk factors (i.e. severity and duration of the disease).

The efficiency of such a method in more complicated scenarios, however, is questionable. For example, what if the analyst would like to take into consideration an important factor of prognosis such as patients' attitude towards treatment? This would necessitate further splitting each state in two more states. With every additional factor, the model becomes increasingly more difficult to handle properly. Would using a Markovian representation make it possible to efficiently consider the key factor of suicidal behaviour? The same reasoning applies: the integration of all relevant factors into a Markov model may render it too complex and prone to bias. Markov models have previously been used to model the cost-effectiveness of relapse prevention interventions for recurrent depression [18-23]. They have also been used as a tool to portray the epidemiology of depression [24-29]. However, we were unable to find any Markov model that simultaneously took into consideration all of the confounding factors just mentioned.

These factors are of great interest to researchers and decision makers alike and, naturally, may merit a more flexible simulation method. Discrete event simulation models may be an opportunity to adequately address the limitations of Markov models, and our intention was to assess the benefits and drawbacks of DES compared with Markov models.

### III.2 Discrete Event Simulation models

Discrete event simulation (DES) is one way of observing the time- dependent (or dynamic) behaviour of a system [30-32]. As a cost-effectiveness tool, DES models have been widely used in various disease areas, including laparoscopic surgery [33], gastric cancer [34], renal diseases [35], drug abuse [36], HIV transmission [37], early breast cancer [38,39] and liver transplants [40]. To our knowledge, DES models have not yet been used within the field of major depression.

Recently, J.J Caro proposed further examination of DES models as a computational tool for cost-effectiveness analyses. In doing so he reiterated the key principles of the method [6]:

####  Entities

Entities are the items that evolve through the simulation. In the clinical simulation of a disease, patients are the entities. The patient is an explicit element of a discrete event simulation model. In DES models, patients are assigned attributes (e.g., age, sex, duration of the disease) with a specific value (distribution) for each. These values are defined at the start of the simulation and may be updated as required: age increases, disease severity levels rise and fall, the number of depressive events increases, etc. Other model specifications such as time horizon and discount rate are encoded in variables. These values may change during the simulation.

####  Events

An event is defined as anything that can happen during the simulation. This can include occurrence of depressive symptoms, remission from depressive symptoms, patients stopping treatment, a suicide attempt, an adverse event, etc. This concept extends well beyond the transitions in a Markov model, because the event need not imply a change in the patient's state. Events can occur sequentially and/or even simultaneously. They can recur – if this corresponds to clinical reality – and they can change the course of a given patient's experience by influencing that patient's attributes and the occurrence of future events. The rates at which events occur can take on any functional distribution supported by the data. They can be dependent on any attributes or variables and these functions can change over time as appropriate.

####  Time

The third fundamental component of a DES is time itself. An explicit simulation clock keeps track of the passage of time. This makes it possible for the analysts to clearly signal the start and end of the simulation and to create secondary clocks that track interim periods such as depression episode duration or remission periods. By making time explicit, a DES enables handling time much more flexibly compared with Markov models because there is no need to define cycle length.

The model described here belongs to the class of models that have been described elsewhere as individual sampling models [41,42]. Rather than following an entire cohort through a model by assigning proportions to different states, discrete event simulation models the pathway of an individual by sampling probabilities from an a priori distribution. This results in greater realism in describing a patient's evolution through the healthcare system and offers more flexibility in the data requirements needed to feed the model. DES models provide an alternative tool capable of considering multiple risk factors and non-Markovian structures (i.e. non memory-less stochastic processes). Peter W. Glynn describes a mathematical formalism for the underlying stochastic process [43,44], named "Generalized Semi-Markov Process" (GSMP). A GSMP is an established formalism for modeling continuous-time stochastic discrete event systems.

Throughout the entire simulation, new information (depending on the triggered events) can be tracked and stored into a temporary variable, so that future events' probabilities can be changed to reflect a patient's new clinical and socio-demographic profile. Patients may then acquire attributes (e.g., a higher risk of relapse) as certain events occur within the model. The attributes of a particular patient influence his/her pathway through the simulation, as well as the economic outcomes associated with the events experienced (e.g., hospitalisation resulting from a suicide attempt).

Thanks to the strength of the assumptions the technique offers, and by modelling individual patient pathways, DES provides a greater degree of flexibility which, when supplied with adequate data, may allow greater confidence in the results [45].

**Figure 6:**
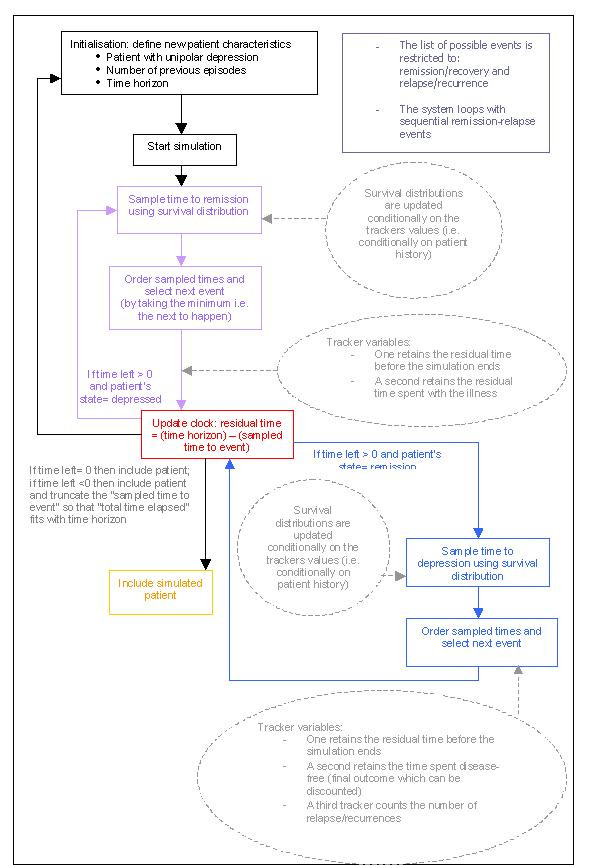
Discrete Event Simulation algorithm.

To examine the properties of DES under practical circumstances, we chose to apply them to the dynamics of depression. The algorithm associated with our problem is depicted in Figure 6. There are no cycle lengths to declare and no health states to define. Disease evolution is pictured using events that will trigger a change of health state. The method chosen to select the next occurring event was that used by Barton et al. [46], the underlying idea being "sample times for each possible event and use the minimum" (the rationale being that once the first event has happened, times to other events may need to be resampled). For each event, therefore, survival distributions were required assuming that no other event was possible. A time was sampled for each event and the earliest time determined which event happened. This is implemented by considering other events as censored events and the other times are discarded.

The information required was survival data conditioned on the number of prior depressive events. The time spent in the "well" state was obtained by summing the tracked sampled times leading to relapse. The time spent in the depressed state was obtained by summing the tracked sampled times leading to remission. Other tracker variables make it possible to count the number of relapses or recurrences occurring during the study period. The patient started the model with a first depressive event (i.e. no prior depressive events). The only event that would remove a patient from the state of depression would be achieving remission of symptoms. Therefore, a "time to remission" was sampled based on the patient's history. The clock advances of the simulated "time to remission" and a test is performed to check if there is time left to continue the simulation (according to the fixed time horizon) or not. Once the patient is symptom-free, he is still subject to relapse. Therefore, a "time to depression" is sampled and the clock is advanced to this sampled "time to depression". If the sampled time was inferior to 24 weeks then the patient was in "simple remission"(i.e., relapse). If the sampled time was superior to 24 weeks, then the patient was in full recovery. Based on these tests, tracker variables count the number of events that occur, and patient's attributes are updated accordingly. Figure 6 displays a graphical representation of the algorithm.

The present DES model reflects a pathway with a very limited list of possible events: there are no competing events. This renders the analysis quite simple and as a practical example makes it possible to visualize the flexibility with which DES models cope with multiple competing events. DES models appear to be a powerful means to address both the problem of a patient's history and the risk for an illness to persist by using survival distributions conditioned by tracker values. Similarly, remission and recovery periods can be easily distinguished by tracking the sampled "time to depression" and see if this sampled time (24 weeks, per consensus definitions) is inferior or superior to a threshold value. If the sampled time to depression was less than 24 weeks, then the patient could not be in full recovery and was, therefore, only in a "remission phase". By implementing various queries, it is possible to define new trackers that will remember whether the patient was completely well or not.

DES models are as efficient as Markov models, with perhaps, slightly more flexibility regarding their implementation. If the analyst wishes to tackle the problem of adherence to treatment (a key factor of interest in modelling depression), DES models are flexible enough to manage this by adding an event to the list of possibilities a patient is likely to experience (along with its own survival distribution) and run the model. The sequence of events experienced by the patient will be randomly generated according to the event selection method previously outlined (i.e. sample "time to events", the first event being the one selected). This means that key events in depression such as attempted suicides, adverse events or any other event for which there is adequate data, can be easily taken into account.

DES models seem to be a promising simulation technique, very flexible and easy to follow for any analyst who may not be familiar either with the key aspects of the disease or with simulation tools in general. DES models are able to overcome Markov model limitations particularly in their ability to take into account multiple events, which can be crucial when trying to depict disease progression as close to reality as possible.

## IV. Discussion

When modelling the course of disease it is important to consider as many disease-specific risk factors as possible in order to provide an informed view of outcomes that may occur. The word 'may' is important because no model can predict any outcome with 100% accuracy. Modelling techniques are evolving in response to criticism aimed at improving their predictive abilities. Discrete event simulation further contributes to the field. While Markov models have served – and continue to serve – the scientific and decision-making communities well, we are of the opinion that DES also offers additional possibilities for modelling patients with depression and their progression through the healthcare system. A frequent criticism of depression models is that they are often too short and, therefore, unable to accurately reflect the true progression of the disorder. In the present case, the DES timeframe was large enough to capture all events occurring during the disease span and even beyond (periods of recovery). As such, distinction between relapses and recurrences (according to whether the patient is experiencing a new episode of depression or not) were shown to be important issues to be taken into consideration in order to more precisely assess the capacity of a given strategy to delay further risk of developing depressive symptoms. The number of previous depressive events, their duration and severity together with patients' adherence to therapy were also proven to be key factors that needed be taken into account in the computational framework.

Despite their lack of memory, Markov models managed to handle the problem of patient history by specifying various health states defined according to risk levels (low, moderate, high risk of relapse). The issue of disease persistence was also addressed, but at the expense of defining multiple temporary health states. A major drawback, however, persists in the handling of multiple events. If analysts truly seek to portray reality as closely as possible, they should consider scenarios that are more complicated and that take into account, for example, suicidal behaviour and patients' attitudes towards treatment. In such situations they may be more likely to employ more elaborate modelling methods such as discrete event simulation.

Health service research in general, and economic evaluation in particular, is commonly associated with a lack of adequate data. In the present case, the intention was to validate the use of a DES model in a conceptual manner, i.e. in terms of its computational validity. To numerically quantify the benefits of DES over Markov models, empirical validation is required. Future research would necessitate comparing simulated results with those obtained from observational data (i.e. perform an external validation). Further research should focus on both the internal and external validity of the conceptual model [47], by collecting adequate data (after systematic review of the literature), then choosing appropriate statistical distributions on parameters, and finally by calibrating the model with reference to results obtained from naturalistic studies. However, when reliable data is not available, DES may be a highly suitable information system that could be used to run a series of different "what if?" scenarios, allowing the user to understand the interaction of the model parameters, and their effects on the output of interest. For example, in the context of exploratory analyses whose purpose would be to identify preferred health outcomes for inclusion alongside a clinical trial, DES may help in defining the requirements of a definitive economic analysis and determine a data collection strategy.

There are certain limits to DES that deserve to be pointed out. First, greater flexibility may only be reached at the expense of supplementary specialist analytic knowledge, which may reduce the evaluator's direct access to the model. Also, it may take time to develop, implement and verify the conceptual model. Moreover, individual-based models like DES models are highly time-consuming, as multiple replications are needed to get good estimates of mean effects. However, variance reduction methods are available that can reduce the number of replications and time needed [48]. Finally, DES may induce over-specification, whereby possible patient pathways become more complex than necessary, thus implying an increase in data requirements.

We deliberately chose here to focus on the methodological aspects of the modelling methods, regardless of the therapeutic strategies and without any costing purpose. However, costing would be equally feasible in both methods: DES models would use variables associated to each event experienced, while Markov models would associate a monetary value to health states.

In order to provide decision makers with a fully specified tool aimed at prioritizing actions for relapse prevention in depression, further work should incorporate, in the form of a DES model, both clinical and economic data in accordance with national and international clinical and pharmacoeconomic guidelines. A practical example of discrete event simulation model for depression, together with judicious distribution choice on parameters (among Weibull, Log-logistic and more generally Gamma distributions) will be the next step of this research, with an aim towards benchmarking results from a DES model with those from standard simulation models.

## V. Conclusion

When considering the practical examples previously presented, discrete event simulation appears to be complementary and appropriate modelling method when applied to depression. Although discrete event simulation has a quite long history in industrial operational research [49-51], it is still not widely used in the assessment of the value of healthcare interventions. DES could provide a comprehensive tool to illustrate the course of depression, thus allowing greater flexibility in depicting the cost-effectiveness of prevention interventions for recurrent depression. In general, the greatest advantages DES has to offer are that it allows the analyst to model more complex and dynamic systems compared with other types of modelling and that it permits experiments that might not otherwise be feasible ("what if?" scenarios) and may provide additional support for expected value of information (EVPI) analyses. The greater flexibility of DES also enables the model to capture more details about the uncertainty in the system being modelled.

## Conflicts of interest

This manuscript is part of the doctoral thesis requirements of Agathe Le Lay, H. Lundbeck A/S provided a grant for this study. Clement Francois and Nicolas Despiegel are employees of H. Lundbeck A/S. Gerard Duru declares no conflict of interest.
